# Treatment of Fistula-In-Ano with Fistula Plug – a Review Under Special Consideration of the Technique

**DOI:** 10.3389/fsurg.2015.00055

**Published:** 2015-10-16

**Authors:** Ferdinand Köckerling, Nasra N. Alam, Sunil K. Narang, Ian R. Daniels, Neil J. Smart

**Affiliations:** ^1^Department of Surgery and Center for Minimally Invasive Surgery, Academic Teaching Hospital of Charité Medical School, Vivantes Hospital, Berlin, Germany; ^2^Exeter Surgical Health Services Research Unit (HeSRU), Royal and Exeter Hospital, Exeter, UK

**Keywords:** complex anal fistula, fistula plug, biological mesh, fistula closure rate, incontinence

## Abstract

**Introduction:**

In a recent Cochrane review, the authors concluded that there is an urgent need for well-powered, well-conducted randomized controlled trials comparing various modes of treatment of fistula-in-ano. Ten randomized controlled trials were available for analyses: There were no significant differences in recurrence rates or incontinence rates in any of the studied comparisons. The following article reviews the studies available for treatment of fistula-in-ano with a fistula plug with special attention paid to the technique.

**Material and Methods:**

PubMed, Medline, Embase, and the Cochrane medical database were searched up to July 2015. Sixty-four articles were relevant for this review.

**Results:**

Healing rates of 50–60% can be expected for treatment of complex anal fistula with a fistula plug, with a plug-extrusion rate of 10–20%. Such results can be achieved not only with plugs made of porcine intestinal submucosa but also those made of other biological or synthetic bioabsorbable mesh materials. Important technical steps are firm suturing of the head of the plug in the primary opening and wide drainage of the secondary opening.

**Discussion:**

Treatment of a complex fistula-in-ano with a fistula plug is an option with a success rate of 50–60% with low complication rate. Further improvements in technique and better studies are needed.

## Introduction

Fistula-in-ano is a difficult problem that physicians have struggled with since the time of Hippocrates (1). Despite the long-standing history of fistula-in-ano and the multiple approaches that are utilized, there is a paucity of high quality data to guide decision (1). In a recent Cochrane review, the authors concluded that there is an urgent need for well-powered, well-conducted randomized controlled trials comparing various modes of treatment of fistula-in-ano (2).Ten randomized controlled trials were available for analyses: there were no significant differences in recurrence rates or incontinence rates in any of the studied comparisons. The American Gastroenterological Association divides the fistula-in-ano into simple and complex (1). Simple fistulas are low – i.e., they involve a small or no portion of the sphincter complex. These fistulas include superficial, low intersphincteric, or low transsphincteric fistula. In addition, communication between the anal canal end skin is only via one tract and is not associated with inflammatory bowel disease, radiation or involve any other organ (1). Complex fistulas are anatomically higher: they involve a significant portion of the sphincter musculature, may have multiple tracts, involve other organs (i.e., vagina) and may be associated with radiation or inflammatory bowel disease. Recurrent fistulas are usually included in this category as well (1).

Fistulotomy, although extremely effective in treating low anal fistulas, is not a feasible option when the fistula tract incorporates a significant amount of the internal and external anal sphincter, as is the case for many high transsphincteric fistulas (3). It is also frequently contraindicated for anterior transsphincteric fistulas in women, for most fistulas in patients with Crohn’s disease, and for fistulas in patients who have diminished continence (3).

The alternative treatment option of a transanal mucosal advancement flap for patients with high transsphincteric fistulas has reported success rates ranging from 59 to 98%. However, these procedures are technically challenging and some authors report incontinence rates of up to 20% (3).

In Crohn’s disease-related high perianal fistulas, the mucosa advancement flap was combined with platelet-rich plasma (4).

Fibrin glue has also been used as treatment option, but with modest or poor success rates of between 0 and 74% (3–8).

Cutting seton procedures result in low recurrence rates, but can cause incontinence in up to 12–25% of patients (3, 9).

Ligation of the intersphincteric tract (LIFT) is a further alternative technique and has been associated with fistula closure rates of between 57 and 94% (3, 9). In a recent systematic review of 26 studies, including only 1 randomized controlled trial and 24 case series, 7 technical variations were used. Primary healing rates ranged from 47 to 95% (10).

Johnson et al. (11) first described the anal fistula plug, a bioabsorbable xenograft made of lyophilized porcine intestinal submucosa.

The following article reviews the studies available on treatment of fistula-in-ano with a fistula plug and calculates the success rates, while paying special attention to the fistula closure rate and the techniques used. The literature reports a success rate ranging from 24 to 88% with the mean follow up of 8 months. A possible explanation for this discrepancy could be differences in patient selection and variation of the technique (5). In a Consensus Conference, it was stated that a frequent issue affecting the plug procedure is a failure in the plug placement technique (5, 12). Therefore, each publication was carefully reviewed to identify the surgical technique employed. This sets this systematic review apart from those published hitherto.

## Materials and Methods

PubMed, Medline, Embase, and the Cochrane medical databases were searched up to December 2014 using the key words: “Anal fistula” A*N*D “Plug,” “Fistula-in-ano” A*N*D “Plug,” “Anal fistula” A*N*D “Fistula plug.” In addition, the references of articles retrieved were searched for relevant articles not previously identified. Sixty-four articles were relevant for this review.

## Results

The first systematic review of the efficacy of a SIS-anal-fistula plug was published in 2010 (13). All randomized/non-randomized, controlled/non-controlled clinical trials, which studied SIS-anal-fistula plug or compared SIS-anal-fistula plug with other ­treatment methods for anal fistula and which reported clinical healing of the fistula as the outcome, were included. Studies on patients with rectovaginal fistula who were treated by SIS-anal-fistula plug and patients undergoing additional procedure (advancement flap or fibrin glue) along with SIS-anal-fistula plug were excluded from the review. One study reporting the usage of an acellular extracellular matrix was not included because the material used was different.

Twelve studies were analyzed in the systematic review (Table 1). These consisted of one RCT (11), seven prospective case series (14–20), and four retrospective case series (21–24). Since the majority of studies analyzed in the systematic review are prospective or retrospective case series, the level of evidence is only 4. Table 2 gives details of the surgical technique used in the studies included in the review.

**Table 1:** **Results of systematic reviews about the efficacy of anal fistula plug in fistula-in-ano**.

Author	Year	Conflict of interest	LoE	Patients	Follow-up	Success rate	Plug extrusion rate
Garg et al. (13)	2010	None	4	317	3.5–12 months	59.9% (range: 24–92%)	18.5% (range: 4–41%)
O’Riordan et al. (3)	2012	None	4	530	3–24, 5 months	54.3%	–
Leng and Jin (25)	2012	NR	2a	167	5.7–14 months	51.5% (range: 20.0–82.82%)	11.1 + 18.9%

**Table 2:** **Surgical techniques used in the studies included in the systematic review of Garg et al. (13)**.

Reference	Surgical technique
Johnson et al. (11)	Self made SIS-anal-fistula plug from a 2 cm × 3 cm SIS – sheet rolled into a conical configuration
	Plug was pulled tip-first into the internal opening
	Suture fixation of the plug at the primary and secondary opening
	Plug was trimmed at the mucosa and skin level
	No complete occlusion of the secondary opening to allow drainage
O’Connor et al. (14)	Tracts were irrigated with hydrogen peroxide
	SIS-anal-fistula plug
	Plug was pulled tip-first into the internal opening
	Excess plug material was trimmed flush with the mucosa and skin
	Suture fixation of the plug at the primary and secondary opening
	Case was taken not to occlude the secondary opening
Champagne et al (15)	Hydrogen-peroxide installation
	SIS-anal-fistula plug
	Plug was pulled tip-first into the internal opening
	Excess plug material was trimmed flush with the primary opening
	Mechanical stability of the plug relies on firmly suturing the head of the plug into the primary opening
	Fixation of the tip of the plug to the edge of the secondary opening
	No complete occlusion of the secondary opening to allow drainage
Ellis (21)	Hydrogen-peroxide installation
	SIS-anal-fistula plug
	No debridement of the fistula tract was performed
	Occasionally, the distal most portion of the fistula tract was opened to ensure adequate drainage
van Koperen (16)	Cleaning with hydrogen peroxide
	SIS-anal-fistula plug
	No surgical debridement
	Remaining portion of the plug was removed
	Plug fixation at the internal and external opening
	The external fistula opening was not completely closed, enabling further drainage from the fistula tract
	Tract was irrigated with polyhexamide solution
Schwandner et al. (17)	SIS-anal-fistula plug
	No currettage, mechanical debridement, or fistulectomy was performed
	Plug was pulled tip-first into the internal opening
	Plug fixation at the internal opening
	The excess plug was trimmed at the mucosa and the former internal opening was covered with mucosa
	Finally, the excess plug material of the external opening was trimmend at skin level, but no further fixation was made
Ky et al. (18)	SIS-anal-fistula plug
	Plug was pulled tail-first into the internal opening
	Excess plug material was trimmed flush at the internal opening with the mucosa
	Plug was sutured deep to the internal opening
	A small mucosal flap was raised as advancement flap over the top of the plug
	Excess material protruding the external opening was excised
	The secondary opening was left open to allow drainage
Lawes et al. (22)	Tract was washed out with hydrogen peroxide
	SIS-anal-fistula plug
	Plug was pulled tip-first into the internal opening
	Excess plug material was trimmed flush with the internal and external opening
	Suture fixation to the mucosa and internal sphincter
Christoforidis et al. (23)	SIS-anal-fistula plug
	Plug was pulled through the internal opening
	Plug was secured at the internal opening
	The excess plug was trimmed of and the rectal mucosa was closed over the plug
	The plug was trimmed flush with the skin
	It was then secured with a stitch on one side of the external opening (15 procedures) or left unsecured (49 procedures)
Thekkinkattil et al. (19)	Tract was irrigated with saline or hydrogen peroxide
	SIS-anal-fistula plug
	The fistula plug was inserted from the internal opening
	The rectal mucosa was closed over the plug at the internal opening along with a deep suture through the internal sphincter
	Special attention has been made so ensure that the external opening was not completely occluded
Garg (20)	SIS-anal-fistula plug
	Plug was pulled through the track from the internal opening
	Any excess plug was cut flush with the internal opening
	The internal opening was then closed over the plug including the submucosa and internal sphincter muscle
	The distal end of the plug was sutered to the side of the external opening taking, care not to occlude it and allow drainage

A total of 317 patients were analyzed in the review by Garg (13) with a follow-up of range 3.5–12 months (Table 1). The SIS-anal-fistula plug procedure had a success rate of *n* = 180/317 (59.9%) ranging from 24 to 92%. The number of complex fistulae reported in 8 out of 12 studies was 186 with a success rate of *n* = 119/186 (64.0%) ranging from 35–87%. In patients with recurrent fistula, the success rate was *n* = 16/34 (47.1%) ranging from 13 to 71%. The success rate in patients with Crohn’s disease was *n* = 26/41 (63.4%) ranging from 29 to 86%. The success rate in patients with single tracts (*n* = 123/184; 66.8%, range 44–93%) seemed better than for patients with multiple tracts (*n* = 21/43; 48.8% range 20–71%). If the patients with plug extrusion were excluded from the analysis, the success rate was *n* = 121/189 (64.0%), ranging from 40 to 90%.The plug extrusion rate was *n* = 43/232 (18.5%), ranging from 4 to 41%.

In 2012, another systematic review was published (3). This systematic review included studies whose results for patients with and without Crohn’s disease could be differentiated. Patients with rectovaginal, anovaginal, rectouretral, or ileal-pouch vaginal fistulas were excluded as were studies where the mean or median follow-up was <3 months.

The systematic review contained 20 studies, consisting of 18 articles and 2 abstracts (26, 27). Among the 20 studies included are two RCTs (28, 29), 10 prospective case series (15, 16, 20, 26, 30–35), and 8 retrospective case series (22, 24, 27, 36–40). Only 5 out of 20 of the publications listed were also included in the review by Garg (13, 15, 16, 20, 22, 24). This systematic review, too, was supported only by level of evidence 4 in view of the predominant number of prospective and retrospective case series.

Table 3 lists the exact surgical technique employed in the studies that were included in the review by O’Riordan (3) and not already analyzed in the Garg (13) review in Ref. (15, 16, 22, 24). Details of the surgical technique are not given for studies for which only an abstract is available (26, 27).

**Table 3:** **Surgical techniques used in the studies included in the systematic review of O’Riordan et al. (3) minus abstracts and studies already analyzed in the review of Garg et al. (13)**.

Reference	Surgical technique
Christoforidis et al. (37)	Fistula irrigated with hydrogen peroxide
	SIS-anal-fistula plug
	Suture fixation of the internal opening
	The excess plug was trimmed of and the rectal mucosa was closed over the plug
	Plug was trimmed flush at skin level and was secured at the external opening in only 30%
Chung et al. (38)Chung et al. (40)	Hydrogen peroxide installation
	SIS-anal-fistula plug
	Excess plug material was trimmed flush with the mucosa at the internal opening and at the external fistula opening at skin level
	Sutures were used to secure The plug to the internal sphincter muscle and to cover the mucosal opening of the fistula
	The external end of the plug was secured to 1 side of the external fistula opening
Wang et al. (39)	Fistula tract irrigation with hydrogen peroxide
	Plug was pulled through internal opening of the fistula
	The plug was then trimmed
	The head of the play was secured to the internal opening by a suture incorporating mucosa, submucosa and internal sphincter
	Closurre of the internal opening of the fistula over the plug
	No fixation of the plug to the external opening
Ortiz et al. (28)	Injection of hydrogen peroxide
	SIS-anal-fistula plug
	Suture fixation of the plug to the internal sphincter
	Closure of the internal opening of the fistula over the plug
	Care was taken to ensure that the external orifice of the fistula was not completely occluded so that the track could drain
	The remaining Plug was cut of the level of the external opening
Schwandner and Fuerst (30) Schwandner et al. (31)	Fistula passage was rinsed with hydrogen peroxide and debrided with a soft-bristle brush
	The external fistula opening was debrided
	SIS-anal-fistula plug
	Insertion Into the fistula through internal opening
	Plug was fixed with several sutures to the sphincter muscle and the inner fistula opening closed
	The external fistula opening was kept open to allow drainage
	Plug was trimmed, but not fixed to the external opening
Zubaidi and Al-Obeed (32)	Curetage and irrigation with hydrogen peroxide
	Plug was inserted through the internal opening
	Excess fistula plug was trimmed from both ends
	Plug was buried into the primary opening using a figure-of-eight absorbable suture, which was inserted deep into the internal sphincter muscle
	At the secondary opening the tip of the plug was tacked to the edge, making sure to not completely occlude the secondary opening to allow drainage of exudates
Adamina et al. (33)	No irrigation
	SIS-anal-fistula plug
	Plug was inserted through the internal opening
	Plug sutured to the internal sphincter
	The tip of the plug was cut at skin level and not sutured to allow drainage
McGee et al. (34)	Irrigation with hydrogen peroxide
	SIS-anal-fistula plug
	Plug was pulled from the internal opening into the fistula
	Excess fistula plug was trimmed from both ends
	The fistula plug was fixed and buried within the internal sphincter at the internal opening
	Avoidance of occluding the external opening
El-Gazzaz et al. (36)	Irrigation with hydrogen peroxide
	SIS-anal-fistula plug
	Pull-through technique from the internal to the external opening
	Fixation to the internal sphincter muscle
	Plug material was trimmed
	Former internal opening was closed deeply with sutures
	Plug material at the external opening was trimmed at skin level
	No further fixation
Lupinacci et al. (35)	Tract washed out with hydrogen peroxide
	Plug was inserted via the primary internal orifice and pulled toward the external orifice
	Plug was cut flush with the anal mucosa
	Plug was anchored With sutures to the internal sphincter
	Plug was carefully covered with anal mucosa
	The external orifice was left open
	Plug was cut again and affixed to the skin
van Koperen et al. (29)	Clearing of the fistula tract with hydrogen peroxide
	Plug was pulled in the tract from the internal opening
	Plug was trimmed
	Plug was sutured in place with of least two sutures
	The external opening was left open to allow for drainage of the tract

The study sample sizes ranged from 4 to 60 patients with a pooled total of 530 patients for this review. Forty-two of these patients had Crohn’s disease, whereas 488 patients did not have Crohn’s disease. The shortest mean or median follow-up in the 20 studies was 3 months, and the longest follow-up was 24.5 months.

Closure of the fistula was successful in 288 of the 530 patients with fistula-in-ano (54.3%; 95% CI 0.50–0.59). The overall success rate for patients with Crohn’s disease was 23 of 42 patients (54.8%), whereas for patients without Crohn’s disease it was 265 of 488 patients (54.3%).

A total of 46 patients experienced plug extrusion (8.7%). Eight of the 20 included articles reported continence levels pre- and post-insertion of the SIS-anal-fistula plug (20, 23, 24, 29, 31, 34, 40). There were no reported cases of any significant change in continence after insertion of the SIS-anal-fistula plug in any of the patients in these studies (*n* = 196 patients).

Leng et al. (25) then published a meta-analysis comparing anal fistula plug vs. mucosa advancement flap in complex fistula-in-ano. The studies included were three RCTs (28, 29, 41), one prospective cohort study (33) and two retrospective case series (37, 38). Hence the level of evidence is 2a. Apart from the RCT by A ba-bai-Ke-re et al. (41), the other studies had also been taken into account in the systematic review by O’Riordan et al. (3) and Garg et al. (13).

The six studies encompassed 408 patients with 167 cases of SIS-anal-fistula plug treatment and 241 with mucosa advancement flap. The difference in the overall success rates and incidence of fistula recurrence was not statistically significant between SIS-anal-fistula plug and mucosa advancement flap in complex fistula-in-ano treatment (risk difference = −0.12. 95% CI: −0.39–0.14; risk difference = 0.13; 95% CI: −0.18–0.43, respectively). However, for the SIS-anal-fistula plug, the risk of postoperative impaired continence was lower (risk difference = −0.08. 95% CI: −0.15–0.02) as was the incidence of other complications (risk difference = −0.06. 95% CI: −0.11 to 0.00). Patients treated with the SIS-anal-fistula plug had less persistent pain of a shorter duration and the healing time of the fistula and hospital stay were also reduced. Another comparative study identified similar results for treatment, in addition to cost savings for the plug-in technique because of the shorter hospital stay (42).

Other studies (43–51), which had not been included in the systematic reviews and the meta-analysis (Table 4) do not have any implications for the results of the systematic reviews.

**Table 4:** **Case series of SIS-anal-fistula plug treatment not included in the systematic reviews and meta-analyses**.

Author	Year	Conflict of interest	Study design	LoE	Patients	Follow-up	Success rate	Surgical technique
Safar et al. (43)	2009	NR	Retrospective case series	4	35	Mean: 126 days	13.9%	Clearing with hydrogen peroxidate
								SIS-anal-fistula plug
								Plug was pulled through the internal opening in the fistula track
								The excess plug is cut and then secured to the internal opening
								The internal sphincter was incorporated into the stitch to have at least mucosa and submucosa covering the plug. The part protruding Through the external opening was trimmed back flush with the skin and an optimal tacking stick was placed
Owen et al. (44)	2010	NR	Retrospective case series	4	32	Median: 15 months	37%	Clearing with hydrogen peroxidate
								SIS-anal-fistula plug
								Plug was drown into the tract from the internal opening
								Internal aspect of the plug was trimmed to length and fixed with sutures
								The overlying mucosa of the anal canal was closed over the internal opening
								The tail of the plug was trimmed to length
Lenisa et al. (45)	2010	None	Prospective case series	4	60	Mean: 13 months	60%	Irrigation with hydrogen peroxide and gentle debridement with an endoluminal brush
								SIS-anal-fistula plug
								Pull-through technique from the internal opening
								The plug was than tightly secured to the internal sphincter muscle
								Excess material was trimmed flush to both openings
								The external opening was left open to drain
Kleif et a. (46)	2011	None	Retrospective case series	4	37	Median: 60.5 days	45.9%	Fistula tract was irrigated with hydrogen peroxide and brushed with a fistula brush
								SIS-anal-fistula plug
								Plug was drown through the fistula tract from the inside opening
								The plug was fixed to the internal sphincter.
								Remaining plug inside was excised and the inner Opening closed with a mucosal flap
								The plug in the external opening was left free of fixation, and sometimes the outer opening was even opened a bit
Chan et al. (47)	2012	None	Prospective case series	4	44	Mean: 10.5 months	50%	Track was flushed with hydrogen peroxide
								SIS-anal-fistula plug
								Pull-through from the internal opening
								Plurg secured at the internal opening by suture including the mucosa and submucosa
								The internal opening was covered by a limited mucosal flap
								Distal end of the plug was trimmed flush with the external end of the opening without fixation
Tan et al. (48)	2013	None	Prospective case Series	4	26	Median: 59 weeks	13.3%	Cleaning of the track with saline and hydrogen peroxide
								SIS-anal-fistula plug
								Pull-through from internal opening
								The plug was secured at the internal opening
								The plug was attached loosely to the skin at the external opening
Cintron et al. (49)	2013	Yes	Prospective case series	4	73	Mean: 15 months	Primary 38% Recurrence 40%	Fistula tract was either gently roughened with a cytette brush or debrided with curette
								Irrigation with hydrogen peroxide
								SIS-anal-fistula plug
								Pull-through-technique from the internal opening
								Plug was trimmed flush with the inner opening
								The plug was anchored to the mucosa/submucosa and internal sphincter
								The plug was completely covered with mucosa
								The end of the plug was then trimmed flush with the external opening
Blom et al. (50)	2014	NR	Retrospective case series	4	126	Median: 13 months	24%	Fistula track was flushed clean with saline or hydrogen peroxide and brushed clean of biofilm
								SIS-anal-fistula plug
								Plug was fixed to the internal sphincter
								Any redundant plug was trimmed of the skin level
								The external opening was excised to secure drainage
Adamina et al. (51)	2014	NR	Prospective case series	4	46	Median: 68.1 months	43.5%	Irrigation of track with saline or hydrogen peroxide
								SIS-anal-fistula plug
								Plug was inserted through the internal fistula opening
								Plug was sutured to the internal sphincter
								The tip of the plug was cut at the skin level and not sutured, left open for drainage

It can thus be stated that treatment of complex anal fistula with SIS-anal-fistula plug is likely to be associated with a failure rate of about 50%. This result is not worse than that obtained for the mucosa advancement flap. However, the plug technique has the advantage of a lower postoperative complication rate and no negative impact on continence. More studies and technical modifications are needed to further improve the plug technique.

For example, Köckerling et al. (52) reported on a modified plug technique in which the extra-sphincteric portion of the complex anal fistula was removed by means of a limited fistulectomy and the remaining section of the fistula in the sphincter muscle was repaired using the fistula plug with fixing button. After a mean of 19.32 ± 6.9 months with a follow-up rate of 77% the success rate was 90%.

Another modification entails the use of plugs made of acellular dermal matrix instead of intestine submucosa (53–56). These are not preconfigured as a plug but are cut out from flat biological meshes. Details of the technique as well as the results are given in Table 5. The studies available show that success rates similar to those achieved with the SIS-anal-fistula plug can also be obtained with plugs made from acellular dermal matrix under similar technical conditions. In comparison to traditional surgical treatment, the fistula recurrence rate was significantly lower in the group treated with acellular dermal matrix (57).

**Table 5:** **Case series of complex anal fistula repair with acellular dermal matrix**.

Author	Year	Conflict of interest	Study design	LoE	Patients	Follow-up	Plug material	Success rate	Surgical technique
Song et al. (53)	2008	NR	Prospective case series	4	30 with low anal fistula	30 days	Human acellular dermal matrix (ADM)	100%	Instillation of hydrogen peroxide
									The plug was cut out with three or four strips
									The ADM – plug was pulled trough from external to internal opening
									The ADM – material was inserted deep to the internal sphincter
									The excess was at skin level
									Care was taken to avoid complete closure of the outer opening to allow drainage. At the end of the procedure, the plug was completely buried within the fistula tract
Hammond et al. (54)	2010	Yes	RCT	2b	26 (two inter-sphincteric, seven mid transsphinteric, four low transsphinteric	Median: 29 months	Porcine acellular dermal matrix, cross-linked (Permacol)	54%	The collagen implant was cut into a strip that approximated the dimensions (width and length) of the fistula tract
									Drawn into position via the inner opening
									Excess material was trimmed at the internal and external opening
									Implant sutured into the tract at both openings
									The mucosa at the internal opening was closed over the tip of the implant
Han et al. (55)	2011	NR	Prospective case series	4	114	Median: 19.5 months	Human acellular dermal matrix	54.4%	Instillation of hydrogen peroxide
									Mechanical debridement with a blunt curette
									A conical biologic plug was fashioned from a
									3 × 5 cm sheet of human ADM
									The plug was pulled tip-first into the internal opening
									The excess plug was trimmed flush with the primary opening
									The plug was sutured deep into the interal sphincter
									ADM material protruding from the secondary opening was trimmed at skin level
									No further fixation
Sarzo et al. (56)	2013	NR	Prospective case series	4	12	Mean: 9.3 months	Porcine acellular dermal matrix	75%	The design of the plug (wedge-shaped with sharp edges) neutralizes the forces of axial displacement and rotation
									Mechanical courettage of the fistular tract was performed
									The device was pulled into the fistula track from the internal opening
									A small mucosal periorificial flap was created
									The plug was then secured to the internal sphincter
									The internal opening was then closed with a mucosa plastic
									The plug was sutured to the external opening
									Finally the external opening was enlarged for drainage

In a pilot study, 10 patients with a median of 3 previous fistula operations were successfully operated on with an autologous cartilage plug from the nose or the ear. The treatment was initially successful in 90% of the patients, but two patients later developed a recurrence (58).

A relative new product for treatment of anal fistulas consists of a synthetic bioabsorbable anal fistula plug composed of a copolymer, from polyglycolic acid trimethylene carbonate, which is gradually absorbed by the body. This plug consists of a button or disc, with numerous tubes attached to it. Depending on the diameter of the fistula canal, several tubes are trimmed. The bioabsorption process is supposed to have been completed after 6–7 months (59). To date, there are only six prospective and retrospective cases series that report on treatment of anal fistulas with this synthetic bioabsorbable anal fistula plug (59–64). The results are illustrated in Table 6. The results obtained for the bioabsorbable fistula plug, too, are very variable, ranging from 15.8–72.7%. As in the case of the biological plug, that may be due to differences in the technical conduct of the operation (Tab. 6) or to differences in patient selection. Otherwise, the results obtained for the synthetic bioabsorbable anal fistula plug are comparable with those obtained for the plug made of biological material.

**Table 6:** **Case series of complex anal fistula repair with synthetic bioabsorbable anal fistula plug**.

Author	Year	Conflict of interest	Study design	LoE	Patients	Follow-up	Success rate	Surgical technique
de la Portilla et al. (60)	2011	NR	Prospective observational study	3	19	12 months	15.8%	The button or disc of the synthetic plug was secured in place at the internal opening with 2 or 3 sutures. The number of tubes was removed based on the estimated diameter. The remaining tubes were sutured together. Tubes were visible at the external opening
Ommer et al. (61)	2012a	yes	Prospective observational study	3	12	6 months	50%	Fixation of the button or disc of the synthetic plug to the sphincter at the internal opening. Coverage of the button by a mucosa flap. Excision of the external opening for better drainage
Ratto et al. (62)	2012	NR	Prospective observational study	3	11	5 months	72.7%	A small submucosal pocket was created around the internal opening. The submucosal pocket was closed including the disc of the plug in the suture. The excess tubes were trimmed of the base of the disc. The prutrading tubes were trimmed 2–3 mm beyond the surface of the perianal skin. The external opening was left open to drainage
Ommer et al. (63)	2012b	yes	Multicenter retrospective case series	4	40	6 months	50%	See Ommer et al. (61)
Heydari et al. (64)	2013	yes	Retrospective case series	4	49	12 months	69.3%	The button or disc was fixed to the mucosa by the use of absorbable sutures. One suture was run through the distal ends of the retained tubes to pull them together. Any tube segments that prutraded beyond the perineal skin were trimmed 1cm over skin level
Stamos et al. (59)	2015	yes	Prospective multicenter case series	3	93	12 months	49%	The button or disc was sutured to the anorectal wall by using at least 3 sutures. Button or disc was not covered by mucosa. The end of the retained tubes was trimmed flush with the skin. No sutures were placed in the external opening, which was left sufficiently open to allow drainage

## Discussion

In summary, healing rates of 50–60% can be expected for treatment of complex anal fistula with a fistula plug, with a plug extrusion rate of 10–20%. That result is not worse than that achieved for the mucosa advancement flap, fibrin glue treatment or ligation of the intersphincteric tract.

The anal fistula plug poses a lower risk of postoperative impairment of sphincter muscle function and other postoperative complications than the transanal mucosal advancement flap. Such results can be achieved not only with plugs made of porcine intestinal submucosa, but also those made of other biological mesh materials, such as acellular dermal matrix, and synthetic bioabsorbable material.

It is possible that additional modifications to the technique, e.g., limited fistulectomy of the extrasphincter portion of the anal fistula, will further improve the outcome. Important technical steps in the successful performance of a complex anal fistula plug repair are a mechanical debridement of the fistula tract or partial removal of the extra sphincteric portion of the tract, pulling the plug tip-first in the internal opening, trimming excess plug material flush with the primary opening, suturing firmly the head of the plug into the primary opening, fixation of the tip of the plug to the edge of the secondary opening and no complete occlusion, but wide secondary opening to allow drainage.

There is a need for more high-quality prospective comparative studies which, in addition to the anal fistula diagnosis, give precise technical details of the operation technique, design and biological or synthetic material of the plugs employed as well as their fixation. Both RCTs and registries lend themselves to that effect.

## Conflict of Interest Statement

The authors declare that the research was conducted in the absence of any commercial or financial relationships that could be construed as a potential conflict of interest.
