# Supplementary material for: Treatment of Fistula-In-Ano with Fistula Plug – a Review Under Special Consideration of the Technique
Source: Front Surg. 2015 Oct 16;2:55. doi: 10.3389/fsurg.2015.00055 (PMC4607815; doi:10.3389/fsurg.2015.00055)
Supplement: Supplementary file 1 [file data_sheet_1.docx]

Appendix

BioMesh Study Group

Ferdinand Köckerling (Chairman), Stavros Antoniou, René Fortelny, Frank A. Granderath, Markus Heiss, Franz Mayer, Marc Miserez, Agneta Montgomery, Salvador Morales-Conde, Filip Muysoms, Alexander Petter-Puchner, Rudolph Pointner, Neil Smart, Marciej Smietanski, Bernd Stechemesser

Aim

The BioMesh Study Group has set itself the task of identifying how best to use biological meshes for the various indications. The first step towards achieving that goal is to compile systematic reviews of the different indications on the basis of the existing literature. The available literature sources will be evaluated in accordance with the Oxford Centre for Evidence-based Medicine-Levels of Evidence (March 2009). Next, based on the review findings corresponding Statements and Recommendations are to be formulated in a Consensus Conference for the use of biological meshes for the different indications. The findings of the Consensus Conference are then to be summarized for a joint publication. This present publication is part of the project undertaken by the BioMesh Study Group.
